# Self-awareness of heart failure in the oldest old–an observational study of participants, ≥ 80 years old, with an objectively verified heart failure

**DOI:** 10.1186/s12877-016-0195-4

**Published:** 2016-01-20

**Authors:** Suzana Selan, Arkadiusz Siennicki-Lantz, Johan Berglund, Cecilia Fagerström

**Affiliations:** Department of Health, Blekinge Institute of Technology, Karlskrona, Sweden; Department of Health Sciences, Faculty of Medicine, Lund University, Lund, Sweden; Division of Geriatric Medicine, Department of Health Sciences, Malmö University Hospital, Malmö, Sweden; Blekinge Centre of Competence, SE-371 81 Karlskrona, Sweden

**Keywords:** Heart failure, Oldest old, Prevalence, Self-awareness, 80+

## Abstract

**Background:**

One of the primary reasons for hospitalisation among elderly individuals with heart failure (HF) is poor self-care. Self-awareness of having HF may be a key-element in successful self-care. The prevalence of self-awareness of HF, and how it is affected by age-and HF-related factors, remains poorly understood. The aims of the present study were to determine the prevalence of self-awareness of HF in participants, ≥ 80 years of age, and to investigate the association between this self-awareness and age-related and HF-related factors.

**Methods:**

A single-centre observational study was conducted in which non-hospitalised participants (80+) with objectively verified HF were identified (*n* = 90). The statement of having HF or not having HF was used to divide the participants into two groups for comparisons: aware or unaware of one’s own HF. Logistic regression models were completed to determine the impact of age-and HF-related factors on self-awareness.

**Results:**

Twenty-six percent (23/90) were aware of their own HF diagnosis. No significant differences were found between the participants who were aware of their own HF diagnosis and the participants who were not. Neither age-nor HF-related factors had influence on the prevalence of self-awareness.

**Conclusions:**

Prevalence of self-awareness of own HF in the oldest old is insufficient, and this self-awareness may be influenced by external factors. One such factor is likely the manner in which the HF diagnosis is relayed to the patient by health care professionals.

## Background

The prevalence of heart failure (HF) increases with age [1] and is as high as 22 % among the oldest old, i.e., people older than 80 [2]. Elderly individuals with HF are high consumers of healthcare, and poor HF-specific self-care is one of the primary reasons for hospitalisation [3]. One of the primary tasks of nurses is to ensure that the person they care for has the ability to conduct HF-specific self-care, and educational interventions conducted by nurses have been proven to improve such self-care in elderly HF patients [4]. However, the components that enable the elderly to engage in this specific self-care have yet to be determined [5].

For elderly individuals to conduct HF-specific self-care, a prerequisite is that the elderly individual is self-aware of having HF, i.e., the patient has been informed about the diagnosis and the information has been understood. Previous studies [6–8] that examined the prevalence of self-awareness of HF among patients over 45 years of age with HF found the prevalence to be between 25-46 %. Halling and Berglund [7] defined HF based on diagnosis by a primary care physician, regardless of if any objective examinations were the basis for the diagnosis or not. The risk is, therefore, that elderly were erroneously included or excluded in that study. Furthermore, Gure and colleagues [6] defined HF through Medicare claims, and, as the authors themselves noted, there is a financial incentive for healthcare systems to report HF-diagnoses to Medicare, even in cases in which an elderly patient has not been diagnosed with HF; i.e., participants in their study may have been incorrectly defined as having HF. Okura and colleagues [8] chose to examine both outpatient and inpatient medical records. A participant in the study by Okura et al. [8] was considered to have HF if the HF diagnosis was validated with two major criteria or one major and two minor criteria developed by the Framingham Heart Study [9]. According to the European Society of Cardiology (ESC) guidelines [10], HF is a syndrome and a diagnosis of it must be based on clinical signs and symptoms (i.e., dyspnoea, oedema or fatigue) in conjunction with an objective verification. Thus, an HF diagnosis should be verified with an electrocardiogram (ECG) and echocardiography or chest X-ray, and preferably also by measuring plasma concentrations of natriuretic peptides. Because none of the above-mentioned studies [6–8] used the ESC guidelines’ recommendations, the potential for selection bias may be present in all three studies. Thus, because of methodological issues in previous studies, some uncertainty remains regarding the prevalence of self-awareness of own HF diagnosis. Because the oldest old is the age group with the highest percentage of HF, this age group should be given priority when self-awareness of own HF-diagnosis is researched.

Self-awareness of having a particular illness is arguably crucial to make adequate health decisions and self-awareness may, therefore, be seen as a part of the individual’s health literacy. Thus, self-awareness should not only be influenced by how health information is communicated by the health care professionals [11], but also by age-related factors, such as cognitive impairment, dependency in daily functioning and depression [12–15]. HF-related factors that could be expected to be related to self-awareness of own HF diagnosis ought to be the severity of the illness [6], i.e., functional capacity in relation to HF-symptoms. There should also be a relationship between the time span linking the date of the HF diagnosis to the date of the questioning, if the patient is aware.

In summary, to conduct HF-specific self-care, a self-awareness of own HF diagnosis needs to be present. There is a lack of studies that have investigated the prevalence of self-awareness of own HF diagnosis in the oldest old with an objectively verified HF, as well as factors associated with this self-awareness. Our hypothesis is that the prevalence of self-awareness of own HF diagnosis is low in the oldest old and that there is a relationship between this self-awareness and age- and HF-related factors. The purpose of the present study was to investigate the prevalence of self-awareness of one’s own HF and the association of age-and HF-related factors in people 80 years of age or older with objectively verified HF.

## Methods

### Population

Study participants were included over a relatively long period of time to build up a sufficient study group and overcome the impact of high mortality in this elderly cohort with HF. Therefore, the sample for the present study was collected from The Swedish National study on Aging and Care-Blekinge (SNAC-B). The SNAC-B study is a longitudinal, population-based cohort study, conducted in a middle-sized municipality in Sweden. At baseline (in 2001), the municipality contained approximately 60 600 inhabitants, and the municipality contains both rural and urban areas. The distribution of age, gender, and functional ability within this sample is similar to that of other rural and urban populations in medium-sized Swedish municipalities [16]. SNAC-B includes ten age cohorts (60, 66, 72, 78, 81, 84, 87, 90, 93 and 96 years), of which the four youngest cohorts were randomly selected and the oldest cohorts included all of the inhabitants in respective age cohorts. Invitation to participate was sent by mail, and those who did not respond to the written invitation were also invited to participate through a phone call. In total, 2 312 community residents were invited and 1 402 agreed to participate in SNAC-B [16]. Data from the SNAC-B study and the other SNAC sites are available after application to the principal investigator for each site. More information about available data and contact information can be found at https://snacsweden.wordpress.com/.

**Fig. 1:**
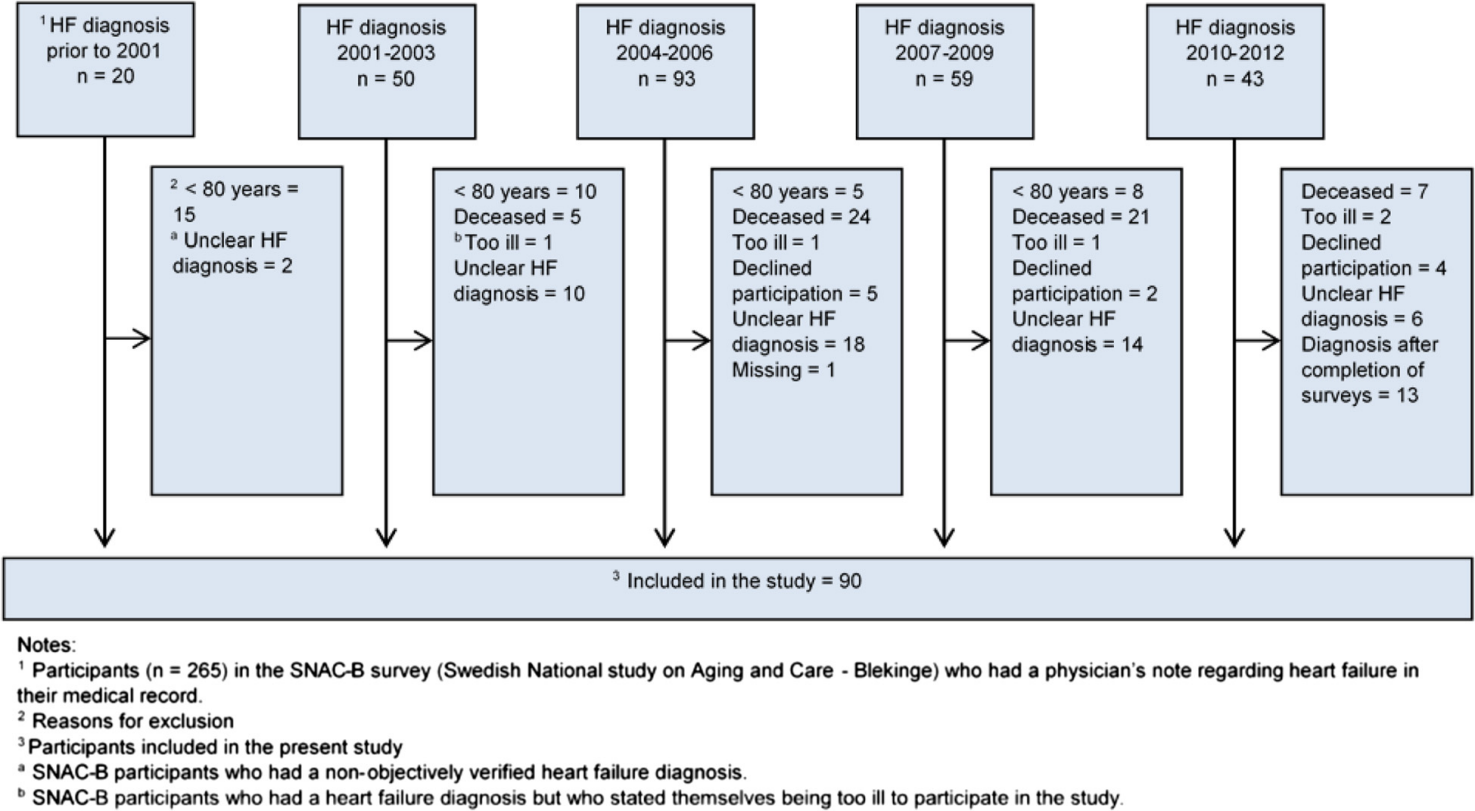
Flowchart of when informants ≥ 80 years old (in 2012) were diagnosed with heart failure (HF) (*n* = 265) and reasons for exclusion

The methodology for the present study has previously been described [17], and implies reviews of inpatient records of those SNAC-B participants who were 80 years and older in 2012 (*n* = 1003) to identify participants with an objectively verified HF. Two hundred-and-forty-one participants had no computerized medical record and were therefore excluded. To be included in our study the participant’s inpatient record should include: (a) notes about HF symptoms (dyspnoea, oedema or fatigue); (b) a pathological ECG; and (c) an objective confirmation of the HF diagnosis by either echocardiography or chest X-ray. For inclusion in our study, the participant also had to be at least 80 years old when diagnosed with HF. Since self-awareness of own HF diagnosis and HF-specific self-care is equally important regardless of housing, participants were included irrespective of whether they lived in their own homes or in a sheltered accommodation. Participants. ≥ 80 years of age, were followed-up every three years between 2001 and 2012, i.e., at four different occasions (Fig. 1).

### Data collection and measurements

After giving written informed consent, the participants included in our study underwent the study examination. The date of the survey and demographic data were collected. If the participant was not able to answer the questions, an immediate family/primary caregiver was asked to assist. Specially trained nurses conducted the surveys at the research centre or in the participant’s own home, which was sometimes preferred to minimise the dropout rate. The study was conducted in accordance with the Declaration of Helsinki [18], and ethical approval was obtained from the Regional Research Ethics Committee at Lund University (LU 605–00, LU 744–00).

### Assessing self-awareness

The participants were asked about HF in the following way: “Have you been diagnosed with HF?” (in the Baseline 2001–03 protocol) and “Have you in the last three years received the diagnosis of HF?” (in the following survey’s protocols). By combining the objective HF diagnosis and the answers on the HF-questionnaire in the survey that immediately followed the HF diagnosis, two groups were identified: one included participants who were aware of their diagnosis, and the other included participants who were unaware of having a diagnosis of HF.

### Age-related factors

#### Cognitive ability

The Mini Mental State Examination (MMSE) measures cognition and has a maximum score of 30. Low scores indicate poor cognitive ability [19]. The cut-off scores suggested by Folstein, Folstein, McHugh, and Fanjiang [20] were used, i.e., > 27 = normal cognition; 21–26 = mild; 11–20 = moderate; and 0–10 = severe cognitive impairment. Due to a small sample size in the groups suffering from moderate and severe cognitive impairment, these two groups were merged.

### Daily functioning

Because the performance of HF-specific self-care often involves complex interaction with the environment, and requires a certain level of cognitive functioning, this self-care is included as part of the instrumental activities of daily living (IADL) [21]. For this reason IADL was chosen to be used when evaluating the participants’ daily functioning. The capacity of participants to perform the instrumental activities: shopping, cooking, cleaning, and transportation were used to describe and compare the dependency in daily functioning. This brief instrument is considered to be sufficient to describe older people’s daily functioning [22]. The participants’ own statement of independence (defined as 0), or dependence (defined as 1) was obtained, and then summed to a total score and categorised according to the following: 0 = excellent daily functioning; 1 = mild impairment; 2 = moderate impairment; 3 = severe impairment; and 4 = total impairment in daily functioning.

### Risk of depression

The Montgomery and Asberg Depression Rating Scale (MADRS) [23] is a 10-item scale rating risk for depression (score-range 0–60). In the present study, a shorter version with nine items of MADRS was used. The question regarding concentration difficulties has a low inter-item correlation [24] and was removed. Snaith et al.’s proposed cut-off criteria for the original version were used, i.e., no risk of depression = 0–6; mild risk = 7–19; moderate risk = 20–34, and ≥35 = high risk [25].

### Heart failure-related factors

#### Functional capacity

New York Heart Association (NYHA) classification system measures functional capacity in relation to HF symptoms [26]. The participants’ own assessment of symptoms when physically active was obtained and were classified as follows: NYHA I = no symptoms during normal physical activity; NYHA II = symptoms (shortness of breath) when climbing a staircase one floor; NYHA III = symptoms when dressing; and NYHA IV = symptoms even at rest.

### Time-span between diagnosis-survey

The time-span between the diagnosis and the following SNAC-B-survey was measured in months and summarized on an individual basis.

### Statistical analysis

Data used for analyses were taken from the SNAC-B survey, which immediately followed a HF diagnosis, and missing data led to exclusion of the specific variable. To avoid low numbers in the cross table, cognition, daily functioning, functional capacity and risk for depression were dichotomised (normal cognition/impaired cognition; excellent daily functioning and mild impairment/moderate, severe, and total impairment in daily functioning; NYHA I/NYHA II-IV; risk for/no risk for depression). Categorisation, absolute numbers and proportions were used to describe the study participants and to determine which of the studied variables could be associated with the self-awareness of own HF diagnosis. Total scores are presented as means with standard deviation (SD) and 95 % confidence intervals (CI) for the mean. Differences between the groups aware/unaware were calculated using the Chi-square test for nominal data and the Mann–Whitney *U* test for interval data. Two logistic regression models (enter) were completed. Model one included only the HF-related factors and model two included both age-related and HF-related factors. Results are presented as odds ratios (OR) with 95 % confidence intervals (CI). The Hosmer-Lemeshow chi-square test was used to assess the models’ goodness-of-fit. To investigate interactions and collinearity among independent variables, tests for Variance Inflation Factor (VIF) were conducted and were found to be acceptable. A *p*-value of ≤ 0.05 was considered to be significant. All statistical analyses were performed using IBM SPSS Statistics for Windows (Version 22.0. Armonk, NY: IBM Corp).

## Results

Out of 265 SNAC-B participants with a HF diagnosis in their inpatient record, 62 (23.4 %) were too ill for inclusion in the study, or died before self-awareness of their HF diagnosis could be established. Another 113 were excluded due to other reasons, detailed in Fig. 1. Ultimately, a total of 90 participants were included in our study. Neither gender differences nor differences in age at HF diagnosis were found between the excluded and the included individuals with HF (*p* = 0.186/0.730).

An average of 18.4 months (SD 14.6) passed between the date of the HF diagnosis and the date of the survey. The average age of the participants at the time of HF diagnosis was 85.9 years old (SD 4.9) and the majority of the participants were women (63.3 %). Physical and mental impairments were common, and 7 (8.5 %) lived in a sheltered accommodation. Impaired cognitive ability was found in 57 (63.3 %) participants, and 17 (18.9 %) were moderately/severely cognitively impaired. Two of these had so profound cognitive impairments that they received help from their spouses to answer the questions in the survey. Almost 30 % had a risk of depression, highly reduced functional capacity (NYHA III-IV) was present in 28 (31.3 %) of the participants, and 30 (33.7 %) had a severe or total need of assistance in their daily functioning.

### Self-awareness of own HF diagnosis

One fourth (23/90) of the participants were self-aware of their own HF diagnosis, and self-awareness of own HF was equally distributed over the different years of the study (*p* = 0.093). Self-awareness of own HF diagnosis was often not present in high functioning participants, i.e., more than one in three participants were cognitively intact (i.e., MMSE total score ≥ 27 out of 30 possible), and 42 % were excellent functioning or only slightly impaired in their daily functioning. And, despite the fact that nearly 60 % demonstrated HF symptoms (i.e., NYHA II-IV), self-awareness of HF diagnosis was infrequent. Participants who were self-aware of their diagnosis were the same age at diagnosis, and they were equally mentally and physically impaired as the participants who were unaware of their diagnosis (*p* = 0.297 to 0.857). Additionally, the time-span between the date of the HF diagnosis and the date of the survey were the same between the two groups (*p* = 0.133). Thus, no association was found between self-awareness of own HF diagnosis and age-related or HF-related factors (Table 1).

**Table 1:** Description and comparison of individuals (≥80 years) with heart failure (HF) who were aware or unaware of this condition (*n* = 90)

	Aware of HF	Unaware of HF	*p*-value^a^
	*n* = 23 (%)	*n* = 67 (%)	
Age Mean (SD)	85.3 (3.9)	86.1 (4.5)	0.484
95 % CI for Mean	63.6 to 87.0	85.1 to 87.2	
Gender			
Male	9 (39.1)	24 (35.8)	0.776
Education			
≤9 years	17 (73.9)	47 (75.8)	0.857
Living arrangements			
Living alone	12 (66.7)	45 (76.3)	0.416
Cognitive ability			0.761
Normal	7 (31.8)	23 (35.4)	
Mild/Moderate/Severe impairment	15 (68.2)	42 (64.6)	
Daily functioning			0.297
Excellent ability/Mild impairment	12 (54.5)	28 (41.8)	
Moderate/Severe/Total impairment	10 (45.5)	39 (58.2)	
Risk of depression			0.309
Absence of risk	15 (65.2)	48 (76.2)	
Mild/Moderate/High risk	8 (34.8)	15 (23.8)	
Time-span between diagnosis-survey			
Months, mean (SD)	14.1 (10.0)	20.0 (15.7)	0.133
95 % CI for mean	9.7 to 18.6	16.1 to 23.9	
Functional capacity			0.373
NYHA I	7 (30.4)	27 (40.9)	
NYHA II-IV	16 (69.6)	39 (59.1)	

Note: The Chi-squared test was used for nominal data. The Mann–Whitney *U*-test was used for interval data

^a^Comparisons between individuals aware of and unaware of own HF

To examine the independent variables’ collaborative influence on self-awareness of own HF diagnosis, two logistic regression analysis were performed. Also in collaboration, the investigated age-and HF-related factors were found to have no impact on self-awareness of own HF diagnosis (Table 2).

**Table 2:** Logistic regression (Enter) awareness of own heart failure HF coded as 1

	Model 1 (*n* = 88)				Model 2 (*n* = 68)			
	OR	*P*-value	95 % CI		OR	*P*-value	95 % CI	
			Lower	Upper			Lower	Upper
Age	-	-	-		0.915	0.306	0.772	1.085
Gender								
Male	-	-	-		0.830	0.838	0.139	4.943
Education								
>9 years	-	-	-		1.938	0.409	0.403	9.315
Living arrangements								
Living Alone	-	-	-		0.393	0.311	0.065	2.396
Cognitive ability								
Normal (reference category)	-	-	-			0.391		
Mild Impairment	-	-	-		2.312	0.273	0.516	10.362
Moderate/Severe	-	-	-		4.467	0.214	0.421	47.445
Activity in daily life								
Excellent ability (reference category)	-	-	-			0.572		
Mild Impairment	-	-	-		2.381	0.366	0.364	15.591
Moderate Impairment	-	-	-		0.654	0.689	0.082	5.221
Severe Impairment	-	-	-		2.532	0.348	0.363	17.651
Total Impairment	-	-	-		0.692	0.761	0.064	7.449
Risk of Depression								
Absence of risk	-	-	-		1.315	0.725	0.287	6.033
Time-span between diagnosis-survey								
Months	0.963	0.083	0.924	1.005	0.966	0.204	0.915	1.019
Functional capacity								
NYHA I	1.627	0.363	0.570	4.647	1.130	0.864	0.278	4.595

Results are presented as odds ratios (OR) with 95 % confidence intervals (CI). In model 1 only HF-related factors were included, while in model 2 both HF- and age-related factors were included

Hosmer-Lemeshow chi-square test: Model 1 = 0.229; Model 2 = 0.625

## Discussion

The most striking result of our study was that only a small percentage of the oldest old were aware of their diagnosis of HF. Our findings are consistent with previous studies [6–8] with different study designs, different age cohorts, and larger study populations. Thus, it appears that most individuals with HF are not aware that they have HF, including the oldest old. However, almost 20 % of the participants were moderately/severely cognitively impaired. This fact should have an impact on the result, especially since only two of the participants had the help of their spouses to answer the questionnaires. However, the logistic regression analysis (Table 2, Model 2) demonstrated that the degree of cognitive functioning did not have any impact on whether self-awareness of one’s own HF diagnosis was present or not. The non-significant results could, however, be a reflection of a small sample size. Nevertheless, the univariate analyses (Table 1) revealed an absence of a tendency for significant findings, and the study would have needed to be up to 30 times larger to reach significance in all of the included variables. Furthermore, even if the small differences in cognitive functioning that were made visible between the groups were significant, the clinical relevance of these findings could be questioned. Additionally, Gure and colleagues’ [6] included approximately 5600 elderly people in their study, and they also found that the degree of cognitive functioning was not related to self-awareness of one’s own HF diagnosis, as was shown in our study. Thus, the degree of cognitive functioning should have a minor impact on whether the oldest old with HF are self-aware of their own HF diagnosis or not, as was shown by our study.

According to Riegel, Dickson, and Faulkner [27], HF-specific self-care can be explained by the theory of naturalistic decision making (NDM). This theory explains human decision making in real life, and depends on the interaction between the decision-making person, the problem and the environment. The authors further reveal how several actors (both the ill person and health professionals) interact in most NDM situations and that the level of interest and commitment can vary between these two actors [27]. The manner in which health professionals provide information about the HF diagnosis (i.e., an environmental factor) can thus affect whether the ill person is aware of the diagnosis or not as well as if HF-specific self-care is conducted or not. Imprecise expressions such as “a bad heart” and “ventricular dysfunction” are often used by health professionals, when informing of HF diagnosis [28, 29], and the prevalence of self-awareness had probably been increased if more imprecise paraphrases of the term “heart failure” had been included in the HF-questionnaire. However, when health professionals use complex terminology, people with HF lose interest in the information provided [29], and the usage of euphemisms when informing patients about HF could be perceived as not recognising the severity of the illness, which may undermine the importance of conducting HF-specific self-care. Thus, the health professionals’ usage of the specific term “heart failure” when informing about the diagnosis should be regarded as an important factor for a successful NDM process and the conductance of HF-specific self-care. This assumption should therefore be further explored.

One of the strengths of the present study is that the study population was drawn from a large, representative, population-based study, with low internal loss of the studied variables. Thus, the results should be considered generalisable to the oldest old with HF. However, despite telephone calls to encourage participation in the study, 73/163 were never examined. Though, only 11 of these dropped out as a result of refusal. The majority (85 %) dropped out because they considered themselves to be too ill or were already deceased (Fig. 1). We chose to study the oldest old with HF, an elderly cohort known with high morbidity and mortality [30]. Consequently, the study’s generalisability to those oldest old who are very old or very morbid at HF diagnosis may be compromised. This, however, underscores the difficulty of studying the oldest old with HF. The chosen study design (to include study participants over a long period of time) has been used before [17], and increased the study population.

Additionally, the participants with HF had their diagnosis objectively verified. This ensures that the participants studied actually had HF. However, the excluded participants who had a non-objectively verified HF diagnosis (20 %) may well have had more HF markers (i.e., higher NYHA classification, higher dependency in daily functioning, etc.). An experienced physician may, therefore, have chosen to diagnose HF without conducting an objective verification, which would have led the participant to be wrongly excluded from our study. It could, therefore, be argued that the results may not be generalisable to individuals with more HF markers. However, the results of our study indicate that HF markers had no association with self-awareness of own HF diagnosis, indicating that the results should be generalisable also to the more seriously ill.

There are probably factors that, in conjunction with the manner in which the information of the HF diagnosis is communicated, can be related to the awareness of one’s own HF diagnosis. The psychological aspect of who/what the individual believes is responsible for their personal health, e.g., Health Locus of Control (HLOC) [31], may perhaps have a relationship with the self-awareness of the presence of HF. Initially, we included HLOC, but due to high internal dropouts, HLOC was later excluded. In our study, HLOC was completed independently by the participant, which may be difficult for the cognitively impaired. Most of our participants were cognitively impaired, which could explain the high internal dropout rate. This fact further illustrates the difficulty in studying the oldest old with HF. However, by the use of the study design presented herein, we managed to establish the relationship between self-awareness of one’s own HF diagnosis and those interpersonal factors that are typically studied in HF trials.

## Conclusions

There is a low prevalence of self-awareness of HF in the oldest old. Factors that could be linked to elderly with HF were found to have no association with this awareness. Thus, external circumstances such as the manner in which the elderly patients are informed about HF, in conjunction with interpersonal factors not covered by the present study, may have a higher impact on the self-awareness of own HF diagnosis than the age- and HF-related factors investigated in this study. This relationship should therefore be further investigated.

## Abbreviations

CI: Confidence intervals
ECG: Electrocardiogram
ESC: European society of cardiology
HF: Heart failure
HLOC: Health locus of control
IADL: Instrumental activities of daily life
MADRS: The montgomery and asberg depression rating scale
MMSE: The mini mental state examination
NYHA: New York heart association classification system
OR: Odds ratio
SD: Standard deviation
SNAC-B: Swedish national study on aging and care-blekinge
